# Continuous Renal Replacement Therapy in Acute Brain Injury

**DOI:** 10.3389/fneph.2022.853677

**Published:** 2022-03-08

**Authors:** Luis Cruz-Llanos, Alejandra Molano, Lilia Rizo-Topete

**Affiliations:** ^1^ Nephrology Service, National Cardiovascular Institute “Carlos Alberto Peschiera Carrillo”, Lima, Peru; ^2^ Renal Therapy Service, Cardioinfantil Foundation, Bogotá, Colombia; ^3^ Department of Nephrology, University Hospital “Dr. José Eleuterio González”, Universidad Autonoma de Nuevo León (UANL), Monterrey, Mexico; ^4^ Department of Internal Medicine, Hospital Christus Muguerza Alta Especialidad, Universidad de Monterrey (UDEM), Monterrey, Mexico

**Keywords:** continuous renal replacement therapy, acute kidney injury, acute brain injury, critical care unit, anticoagulation

## Abstract

Acute brain injury is the sudden and reversible loss of brain self regulation capacity as a disruption of the blood-brain barrier that conditions metabolic and inflammatory disorders that can exacerbate acute kidney injury in a critical setting; specifically it has been described that the alterations of the internal environment that come from the severity of the acute kidney injury increases the risk of endocranial hypertension and cerebral edema; in this context, injuries should be identified and treated in a timely manner with a comprehensive approach. Continuous renal replacement therapy is an extracorporeal purification technique that has been gaining ground in the management of acute kidney injury in critically ill patients. Within its modalities, continuous venous venous hemofiltration is described as the therapy of choice in patients with acute brain injury due to its advantages in maintaining hemodynamic stability and reducing the risk of cerebral edema. Optimal control of variables such as timing to start renal replacement therapy, the prescribed dose, the composition of the replacement fluid and the anticoagulation of the extracorporeal circuit will have a significant impact on the evolution of the neurocritical patient with acute kidney injury. There are limited studies evaluating the role of hemofiltration in this context.

## Introduction

Acute brain injury (ABI) is the sudden and reversible loss of brain autoregulation capacity as a consequence of a disruption of the blood-brain barrier (BBB) ​​that conditions metabolic and inflammatory alterations that lead to cerebral edema and increased intracerebral pressure (ICP) (1). ABI is classified mainly as: traumatic, which results from forceful or penetrating mechanisms, and non-traumatic, the most representative entity of which is the ischemic or hemorrhagic cerebrovascular accident. In any of these scenarios, the patient requires to be managed in a critical care unit.

Studies in patients with traumatic brain injury and aneurysmal subarachnoid hemorrhage have shown that about 89% develop dysfunction of at least one non-neurological organ and this correlates with the severity of brain damage (2, 3). Among the most frequent complications are respiratory failure, heart failure and hematological alterations, with a limited description of alterations in renal function in this context (3).

The effect of ABI on kidney function is attributed to changes in the management of natremia, with hyponatremia being the most frequent hydro electrolytic alteration in neurocritical patients, a consequence of the syndrome of inappropriate secretion of antidiuretic hormone (SIADH) or of the cerebral salt-wasting syndrome as a result of the increase in natriuretic peptides that may occur post acute subarachnoid haemorrhage and pituitary surgery. Likewise, the activation of the visceral sympathetic system in patients with ABI produces systolic hypertension that can reduce renal blood flow due to myogenic effect with increased tubular sodium reabsorption (4, 5).

On the other hand, the systemic inflammation that occurs after traumatic ABI generates functional alterations and apoptosis in renal tubular epithelial cells that can lead to subclinical acute kidney injury (AKI) (6).

The majority of patients with traumatic ABI are young with previous normal kidney function; however, even in them an incidence of acute kidney injury is reported that ranges between 8% and 23% with significantly increases the risk of morbidity and mortality, with a higher incidence of tentorial herniation being described (2, 4, 6–8).

On the other hand, ischemic and hemorrhagic stroke occurs in older patients with multiple comorbidities, and the incidence of AKI in these patients is 14% and 21% respectively, showing higher mortality in patients with ischemic stroke (9). The etiology of AKI in this cohort is multifactorial, the most prevalent being systemic tissue hypoperfusion, sepsis, and the use of nephrotoxic agents (10, 11).

**Figure 1:**
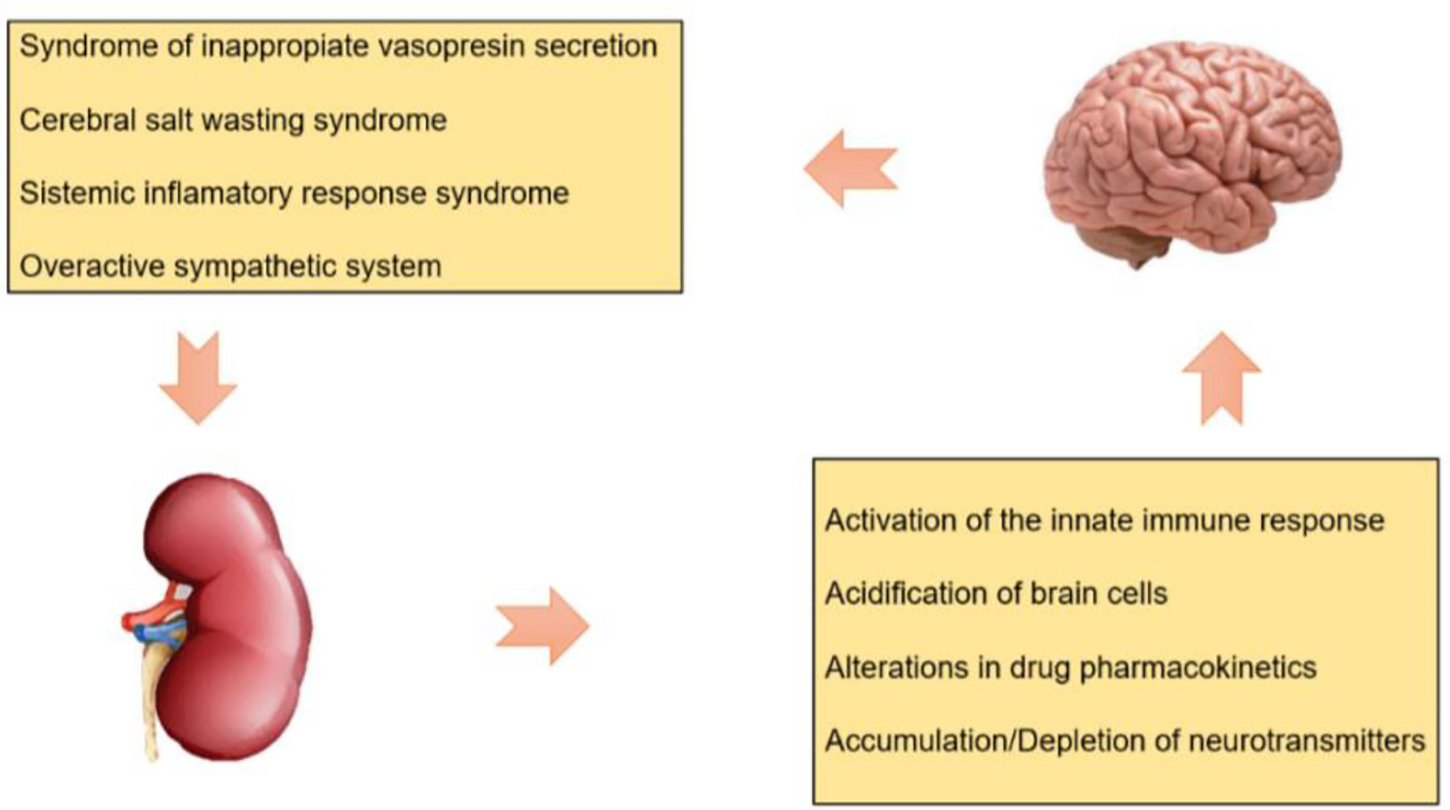
Brain - kidney axis: pathophysiological interaction.

The severity of AKI has deleterious effects on the integrity and permeability of the BBB that exacerbate intracerebral hypertension through the following mechanisms (**Figure 1**):

### Activation of the Innate Immune Response

Ischemic AKI is associated with increased production and decreased clearance of proinflammatory cytokines, tumor necrosis factor alpha (TNF α) stimulates the sympathetic nervous system and leads to reduced cerebral blood flow and might contribute to cytotoxic brain edema by mediating expression of type 4 aquaporins (AQP4) on astrocytes. Moreover, has been reported upregulation of toll like receptor type 4 (TLR-4) in the hippocampus causing local inflammation, leads to symptoms of cognitive impairment in patients with AKI (4, 12–14).

### Acidification of Brain Cells

Metabolic acidosis secondary to AKI activates acid-sensing ion channels, resulting in cellular influx of sodium and calcium. This leads to cell membrane depolarization, cellular injury, and potentially cell death. Brain intracellular acidification leads to oxidative deamination of glutamate through glutamate dehydrogenase and thus altering neurotransmitter balance (4, 14).

### Alterations in Drug Pharmacokinetics

AKI is associated with downregulation of organic acid transporters (OAT) at the renal tubular level (OAT 1, OAT3) and at the brain level (OAT3), this lead to reduction in uraemic toxin renal excretion and reduces the efflux of drugs and organic solutes from the brain, respectively, leading to increasing the risk of drug accumulation and toxicity (4, 14).

### Accumulation/Depletion of Neurotransmitters

An interference with the transporter of L arginina (CAT1/SLC7A1) during AKI may both potentially cause the accumulation, as well as the depletion of amino acids and neurotransmitters within the brain (14, 15).

Therefore, early diagnosis and management of AKI is considered pertinent in order to reduce the risk of in-hospital mortality.

## Role of Nephrology Interventions in ABI

The management of patients with AKI and ABI is based on a comprehensive approach with standard conservative measures such as ventilatory support, neuroprotection and adequate treatment of the hemodynamic state with fluids and vasopressors that allow maintaining the mean arterial pressure and with it the cerebral and renal perfusion pressure.

An important pillar in the management of AKI once established is renal replacement therapy (RRT) in a timely manner. Among the modalities of regular use in critical care unit, we can highlight conventional intermittent hemodialysis (HD), sustained low efficacy daily dialysis (SLEDD) and continuous renal replacement therapy (CRRT), which do not differ significantly in terms of to the mortality rate in these patients; however, the evidence suggests a greater benefit of hybrid and continuous therapies given their better hemodynamic tolerance with a decrease in the risk of brain edema, which is especially relevant in patients with ABI (16, 17).

After ABI, patients are at risk for cerebral edema, elevated ICP, and cerebral ischemia due to breakdown of the blood-brain barrier (vasogenic edema) and disrupted cerebral blood flow autoregulation that may be exacerbated in HD by several mechanisms: the idiogenic osmole hypothesis, reverse urea effect, and the rapid exchange of bicarbonate frequently seen shortly after initiation of HD and preceded by hypotension, which lowers cerebral perfusion pressure (CPP) which in turn may increase ICP by compensatory cerebrovascular vasodilatation with the formation of additional idiogenic osmoles even in non-trauma patients. Several trials have demonstrated increases in intracranial pressure in chronic HD patients (18, 19). Because of that and the more stable hemodynamic profile with continuous modalities of RRT, CRRT has become the preferred mode (20). Main indication of CRRT is to correct acidosis and inflammatory mediators present in AKI. There is still controversy regarding the modality of CRRT and optimal timing of initiating interventions for survival benefit (20–22). Discontinuation of CRRT initiates when renal function is reestablished. Is a matter of controversy the unified criteria to define renal recovery but must be individualized according to urine output and acid- base and hydro- electrolytical status (21).

CRRT can be applied under four modalities that meet the substrate of all the biophysical principles of hemodialysis: Continuous slow ultrafiltration (SCUF), continuous venovenous hemofiltration (CVVH), continuous venovenous hemodialysis (CVVHD) and continuous venovenous hemodiafiltration (CVVHDF), the which should be prescribed in a dynamic and individualized way according to the clinical characteristics of the patient and therapeutic objectives, and can be established sequentially (17, 23).

CVVHD is based on diffusion whereby blood flows through a semipermeable membrane against a sterile dialysate solution with flow in the opposite direction. Solute exchanges depending on the molecular size of the solutes, concentration gradients, membrane cut-off and exchange duration. Diffusion is more efficient in the clearance of small-molecular-weight substances (less than 500 Daltons e.g. K +, Ca 2+) (17).

CVVH is based on convection whereby a pressure gradient is generated across a semipermeable membrane and plasma water and solutes are removed through the pores of the membrane. To compensate for unwanted losses of fluid and electrolytes, replacement fluid is administered. Convection is more efficient at the clearance of large molecular- weight substances (500–5,000 Daltons e.g. cytokines) and is usually prescribed in inflammatory conditions based in the clearance (e.g. sepsis and post cardiovascular surgery) (17). However, scientific evidence has not shown significant differences between these two techniques in relation to mortality, hospital stay and renal recovery in critically ill patients (17, 20).

In AKI, urea and other solutes as circulating cytokines are increased. In ABI, those solutes pass into the brain because the blood-brain barrier is disrupted. This influx is initially compensated by astrocytes taking up additional ions and water. However, this compensation mechanism is limited and thereby cerebral edema (cytotoxic edema) can become worse. As CRRT, mainly convective modalities removes gently fluid, solutes, and inflammatory cytokines, CVVH has been postulated as beneficial in patients with intracranial hypertension (18).

**Figure 2:**
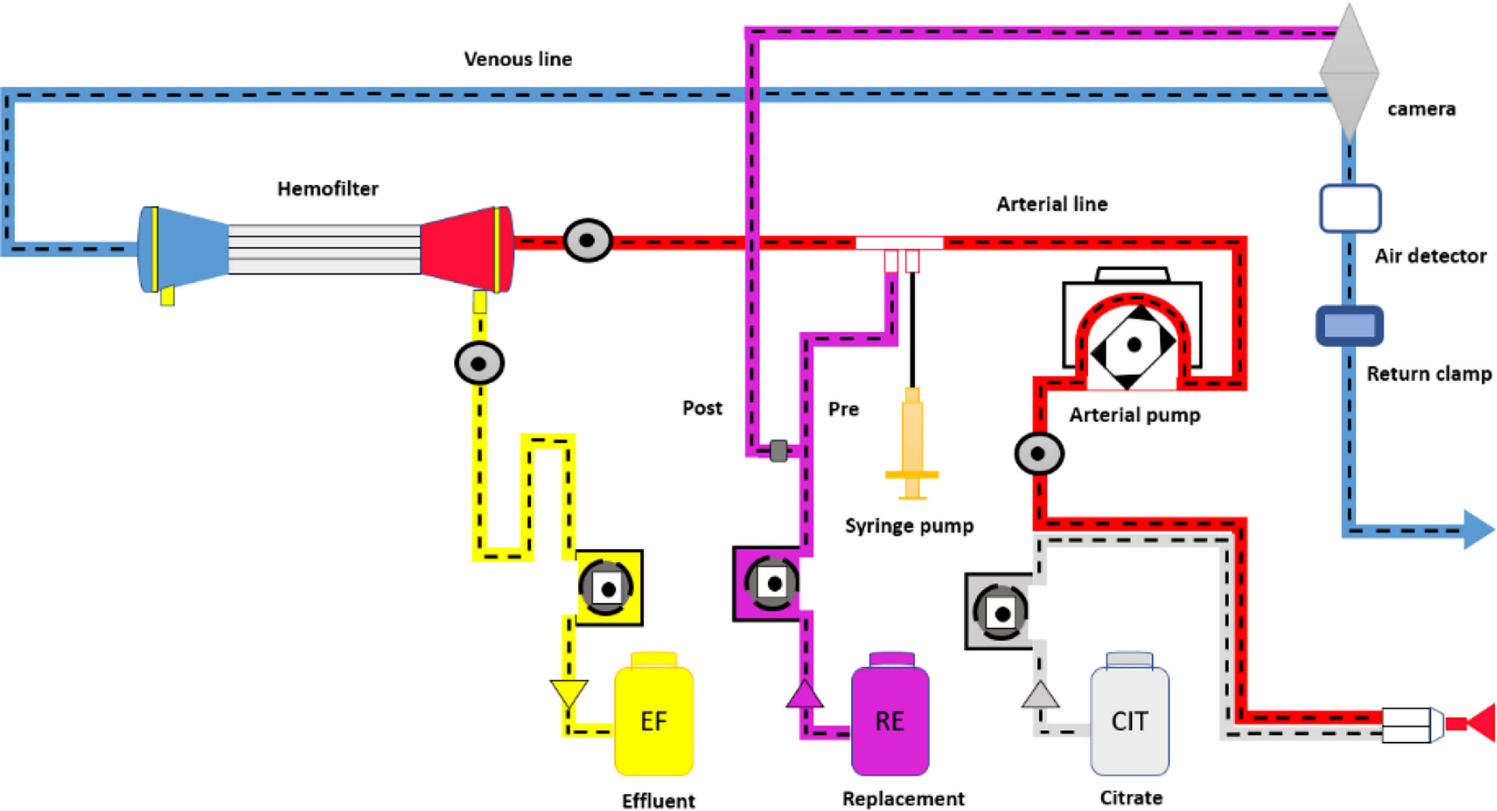
Continuous venous hemofiltration process.

One of the most widely used modalities in the context of critical care patients is CVVH, this technique allows a gradual reduction in the concentration of uremic toxins and fluids, avoiding sudden changes in serum osmolality and favoring hemodynamic stability, advantages that give it superiority over intermittent hemodialysis in the context of the patient with ABI. There is no enough evidence to recommend any CRRT modality over other, but physiologically, convective therapies may result more efficient (17, 20, 21, 23, 24). **Figure 2** shows the CVVH process.

## Discussion

The loss of physiological cerebrovascular autoregulation experienced by the patient with ABI results in a loss of the linear relationship between brain volume and ICP becoming exponential, meaning that a small increase in volume will induce a major increase in intracranial pressure (Langfitt curve). Moreover, the variables such as cerebral blood flow and CPP will depend directly on the mean arterial pressure and of cardiac output, hence the relevance of maintaining hemodynamic stability during CRRT (25, 26).

CVVH complies with the hemodynamic profile due to the removal of pro-inflammatory cytokines and myocardial depressing factors, as well as an increase in the concentration of vasoactive peptides such as endothelin 1 that exerts its vasoconstrictor effect during therapy (21).

By using CRRT, the aim is to prescribe an delivered dose of therapy to patients, especially in terms of acid-base balance, electrolyte balance and hemodynamic stability, which are cornerstones of the survival of patients in critical care, being decisive in ABI.

The effluent flow is an estimation of CRRT dose with several limits, appropriately mentioned by the authors below. The Kidney Disease Improving Global Outcomes (KDIGO) group recommends a EFV of 20 - 25 ml/Kg/h (regardless of the modality and form of pre or post filter fluid replacement); however, since there is a difference between the effective dose and the prescribed dose (related to circuit pauses), a dose of 30 - 35 ml/Kg/h is proposed to achieve the proposed objective that has been related to better outcomes (27–29).

The delivered dose in CVVH depends on several factors such as blood flow through the vascular access, the effective time of treatment, the preservation of the extracorporeal circuit through adequate anticoagulation and the location of the replacement fluid (in the case of using pre filter, decreases solute clearance by 30 - 40%). Tools have been defined to measure the adequacy of the 0CRRT, however, there are still challenges to establish the control of the prescribed dose in this scenario, the four measurements of the quality of the dose in CRRT are: the clearance administered, the ratio of effective to prescribed dose, therapy time, and solute control (27).

In terms of time of initiation of CRRT, there is lack of evidence in AKI, even more when associated to ABI. Several studies have suggested that strategies for starting RRT early (2 - 7.6 hours after randomization) vs late (31 - 57 hours after randomization) do not differ significantly in mortality or RRT - free days in critical units; however, neurocritical patients were not included in these studies (30–33). Some trials recommend early start to ameliorate the osmotic effect of cytokines, nonetheless, the majority of published data includes AKI patients with classical indications for CRRT (20). Some animal models postulate that CVVH even without AKI may result of benefit in ABI (34). The ELAIN study included a small number of neurosurgical patients and although it suggests that the early strategy (8 hours after diagnosis of 2 AKI) reduces mortality over the first 90 days compared with delayed initiation of RRT (12 hours of stage 3 AKI) (35), more clinical research about CRRT timing is needed to establish conclusions.

One of the factors that significantly impacts in the effectiveness of CRRT is the anticoagulation of the extracorporeal circuit, since the critical patient presents a systemic inflammatory state that gives rise to a misbalance between the level of procoagulant and anticoagulant proteins associated with endothelial dysfunction causes a prothrombotic state. Anticoagulation of the extracorporeal circuit can be performed with unfractionated heparin or low molecular weight heparin, complying with strict dosing protocols that prevent bleeding complications. In the context of the patient with ABI, alternative anticoagulation regimens should be chosen, given the high risk of intraparenchymal, subdural, or subarachnoid hemorrhage potentially associated with heparin-induced thrombocytopenia (28, 36).

The KDIGO guidelines propose regional citrate anticoagulation (RCA) as an alternative method. In fact, it is recommended in the management of CRRT patients over other strategies, especially in patients with a high risk of bleeding, such as those with ABI (29).

Citrate is a 191 Dalton negatively charged small molecule that exhibits a chelating action on divalent ions (Ca and Mg), inhibiting the calcium-dependent coagulation cascade. In addition to its anticoagulant effect, it is attributed a reduction in leukocyte activation, reducing the inflammatory reaction (37).

The most commonly used presentation is that of trisodium citrate and it is described that the objective of the citrate concentration in the circuit must range between 4 - 6 mmol/L to achieve an ionic calcium concentration <0.4 mmol/L, a threshold that provides the anticoagulant effect, prolonging the half life of the extracorporeal circuit and reducing the risk of major bleeding (28, 36).

Given its mechanism of action, the therapeutic use of citrate involves the risk of disturbances of acid-base and electrolyte balance (metabolic alkalosis, hypocalcemia, hypomagnesemia, hypernatremia) and since its metabolism (liver, muscle, and kidney) produces bicarbonate in a 1:3 ratio. Furthermore, it has been described that exogenous citrate reacts with carbonic acid and gives rise to sodium bicarbonate, which contributes to the development of metabolic alkalosis (36–38).

Other anticoagulants have been described such as Nafamostat mesylate (NM), a synthetic serine protease inhibitor, has been used in hemodialysis patients at a high risk of bleeding because of its short half-life. NM is a safe and effective anticoagulant for CRRT and allows sufficient filter survival without increasing the risk of bleeding in critically ill patients with AKI and bleeding tendencies (39). Nafamostat has anti-inflammatory and endothelial protective effects; furthermore, studies have shown a neuroprotective role during ischemia-induced brain injury *via* the inhibition of thrombin expression and activity in the brain, particularly in neurons, which suggests that NM could be a potential drug for the treatment of ischemic stroke patients (40, 41).

The patient with ABI requires strict control of some variables of the CVVH such as the composition of the replacement fluid to reduce risk of complications (42). The use of acetate-free replacement solution is recommended as it allows rapid control of metabolic acidosis and uremia, this technique is based on the separate infusion of water and electrolytes in the pre-filter and on the administration of post-filter sodium bicarbonate (43).

The sodium balance is very important to avoid complications in neurocritical patients and to select the correct concentration of sodium in the replacement solution, the ultrafiltrable sodium value must be taken into account. The use of supraphysiologic sodium concentrations is suggested to maintain hemodynamic stability and prevent the increase in intracerebral pressure; however, commercially available replacement solutions used for CRRT contain a sodium concentration of 140 mEq/L, which is lower than the desired 150 - 155 mEq/L serum concentration needed to induce a hyperosmolar state sufficient to counteract the “reverse urea effect”. The effective final sodium concentration delivered to our patients on CRRT is also affected by the rate of delivery of at least 3 solutions: Anticoagulant Citrate Dextrose Solution-A (ACD-A) for RCA with a sodium concentration of 224 mEq/L infused as pre-pump fluid, other base intravenous fluids with 0.9% saline (154 mEq/L) used for medications delivered peripherally and hypertonic saline solution with 3% saline (513 mEq/L of sodium) which can be infused by continuous infusion peripherally preferably (44–46).

In patients with chronic severe symptomatic hyponatremia and concomitant AKI who require CRRT and have risk conditions for osmotic demyelination such as traumatic brain injury, the increase in serum sodium should be gradual, with a daily rate of increase of 4 - 6 mEq/L (47). When using CVVH, the serum sodium correction rate can be controlled by making successive dilutions of the replacement fluid bags every 24 hours. Simply, the sodium of the replacement fluid needs to be about 3-4 mEq/L higher than the desired goal serum sodium while delivering a EFV of 30 ml/Kg/h every 24 hours. The replacement fluid’s sodium could be reduced by adding sterile water to standard replacement fluid bags. Alternatively, sterile distilled water could be exchanged for a volume of replacement fluid (44, 48).

Within the alterations of divalent ions, hypocalcemia can cause neurological disorders manifested as tonic-clonic seizures and since 60% of calcium total plasma is ultrafiltrable, a calcium concentration of 3 mEq/L in the replacement solution is recommended, this is especially relevant in the use of RCA (49).

In a critically ill patient such as the patient with ABI, the rest of the CRRT modalities can also be used according to the clinical condition of the patient (oliguria, sepsis, uremia) according to the days of evolution and progress remembering that it is a dynamic therapy in which the progression of modalities can be carried out as necessary, either ultrafiltration (SCUF) or hemodialysis (CVVHD) and if necessary, use all modalities together as it would be CVVHDF when diffusion and convection are required to offer benefit to these patients.

## Conclusion

Critically ill patients with AKI present metabolic, electrolyte, inflammatory and hemodynamic alterations that exacerbate ABI and vice versa, so timely management could modify the course of its evolution. In this sense, the CVVH fulfills the appropriate profile to limit the pathophysiological interaction; however, prospective studies with a cohort are necessary to assess its impact on mortality.

## Author Contributions

LC has the original idea, collect and order the bibliography and information. LC and LR made the design of the review article and wrote the text. AM and LR made the final revision and paper the paper for publication. All authors contributed to the article and approved the submitted version.

## Conflict of Interest

The authors declare that the research was conducted in the absence of any commercial or financial relationships that could be construed as a potential conflict of interest.

## Publisher’s Note

All claims expressed in this article are solely those of the authors and do not necessarily represent those of their affiliated organizations, or those of the publisher, the editors and the reviewers. Any product that may be evaluated in this article, or claim that may be made by its manufacturer, is not guaranteed or endorsed by the publisher.
